# O8-2 Effects of the HEPA Oblomov methodology on primary school children

**DOI:** 10.1093/eurpub/ckac094.058

**Published:** 2022-08-29

**Authors:** Nicolas Franck, Maurine Remacle, Marc Cloes, Alexandre Mouton

**Affiliations:** Sport Sciences, University of Liege, Liege, Belgium

**Keywords:** HEPA, Physical education, health education, school, intervention

## Abstract

**Background:**

If children are encouraged early in childhood to engage in a physically active lifestyle and if they are used to practice physical activity (PA) as children, they are likely to be more active as they grow up (Telama et al., 2014). Nevertheless, in Belgium, only 2% of children 6 to 9 years of age are able to reach PA international recommendations (Wijtzes et al., 2016). Fortunately, holistic school-focused initiatives can raise children's level of physical activity (Heath et al., 2012). Accordingly, the aim of the study was to scrutinise the influence of an innovative pedagogical approach on self-reported PA and lifestyle habits among elementary school children.

**Methods:**

176 pupils (11-13) and 5 physical education (PE) teachers were recruited in the area of Liege. Those pupils took part in a 10 weeks intervention including one weekly session of PE. PE lessons are original since they combine High Intensity Interval Training (HIIT), dramatization and health education. Assessments were performed before (T0), during (process analysis) and after the intervention (T1). Childrens' levels of PA were assessed with the PAQ-C, as their food habits were assessed with the Adolescent food habits Checklist. Children were also invited, before each session, to notify and share their good practices related to the health education activities proposed during the lesson.

**Results:**

Results exposed significant improvements in self-reported PA (3,09 to 3,26 scores; p > 0,000). As we sort out results by gender, we noticed higher improvements among girls. In contrast, we observed a slight and non-significant decrease in children's food habits (13,88 to 13,55 scores; p = 0,24). On over 400 good practices collected, half were related to physical activity and hydration. By involving physical and psychosocial objectives, the study is expected to provide a deeper understanding of the impact of this teaching method on children.

**Conclusion:**

As Oblomov pedagogy seems to have the potential to generate health basic knowledge as well as pleasure of being physically active, it could be extended to other various settings such as obesity prevention. This method will also be shared with PE teachers in Belgium, as part of the PE curriculum reform in Belgium.

